# Transcranial direct current stimulation in individuals with severe traumatic brain injury in the subacute phase: a case series

**DOI:** 10.3389/fnhum.2025.1552387

**Published:** 2025-04-25

**Authors:** Barbara Naeme de Lima Cordeiro, Jader Vinicius Da Silva Rocha, Elizangela Kuster, Aurore Thibauth, Lucas Rodrigues Nascimento, Chad Swank, Guilherme Peixoto Tinoco Arêas, Walter Gomes da Silva Filho, Carolina Fiorin Anhoque, Wellingson Silva Paiva, Jéssica Costa Buarque, Fernando Zanela Arêas

**Affiliations:** ^1^Neurorehabilitation and Neuromodulation Laboratory, Federal University of Espirito Santo, Vitória, Brazil; ^2^Coma Science Group, GIGA Consciousness, University of Liège, Liège, Belgium; ^3^Baylor Scott & White Research Institute (BSWRI), Dallas, TX, United States; ^4^Physiological Sciences Laboratory, Federal University of Amazonas, Manaus, Brazil; ^5^Division of Neurosurgery, Hospital das Clinicas, University of São Paulo, São Paulo, Brazil; ^6^Neurorehabilitation and Neuromodulation Laboratory, Federal University of Espirito Santo, Vitória, Brazil

**Keywords:** neuromodulation, traumatic brain injury, tDCS, reahabilitation, case report

## Abstract

The aim of this study is to report clinical cases of patients with severe traumatic brain injury (TBI) who underwent transcranial direct current stimulation (tDCS) in the subacute phase. We hypothesize that tDCS will improve the functional and cognitive recovery of patients. 5 men, admitted with severe TBI, and Glasgow Coma Scale (GCS) score ≤ 8 on admission or at some point during hospitalization, were in the subacute phase of the trauma (between 2 and 16 weeks). Participants received 5 sessions of tDCS every day. The results were measured at the beginning and at the end of the 5 sessions. The application of tDCS with an active electrode (anode) was applied to the region of the left dorsolateral prefrontal cortex (LPFC - F3) and the cathode was positioned over the contralateral supraorbital area. Clinical outcomes were measured through cognitive assessment, Mini Mental State Examination (MMSE), mental health and depression, Hospital Anxiety and Depression Scale (HADS-A), pain, visual analogue scale (VAS), Functional Independence Measure (FIM), Rancho Los Amigos Scale (RLAS) and Glasgow Outcome Scale - Extended (GOS-E), were applied to classify the patient’s condition. For characteristics of participants and findings results, descriptive statistics were presented as mean ± Standard Deviation (SD). The results after the tDCS intervention show substantial improvement in the assessed. The research demonstrates the potential benefits of using tDCS in patients with TBI, but also provides a practical basis for applying these techniques in clinical settings.

## Introduction

Traumatic brain injury (TBI) is a global health problem and has been one of the main causes of morbidity, disability and mortality, especially in adults young people. Worldwide, more than 50 million individuals suffer a TBI each year (Bruns and Hauser, 2003). In Brazil, it is an important public health problem due to the high rate of disability in the young population, causing these individuals to interrupt their professional activities early (Areas et al., 2019). Damage to neuronal tissues associated with TBI falls into two categories: (I) primary injury, which is caused directly by mechanical forces during the initial insult; and (II) secondary injury, which refers to the cascade of cellular and molecular processes initiated by the primary injury. The immediate impact of different mechanical traumas to the brain can cause two types of primary lesions: focal and diffuse brain lesions. Axonal injury is the most common consequence of diffuse trauma, accounting for approximately 70% of cases TBI cases. TBI is complex and dynamic and results in changes in the function and structure of practically all elements of the brain. A proportion of survivors of severe TBI, after prolonged hospital care, require special attention and may experience disorders long-term physical, cognitive and psychological^.^ Among the disabilities, cognitive dysfunction is a consequence of brain injury that affects a large proportion of those who survive after moderate to severe injury (Newcombe et al., 2011)^.^ A scale widely used during the initial examination to estimate the severity of TBI is the Glasgow Coma Scale, which consists of a quick and reproducible scoring system. A score of 13 to 15 indicates mild TBI; 9 to 12 moderate TBI and 8 or less severe TBI.

Non-invasive brain stimulation (NIBS) techniques have shown potential as therapeutic options for neuropsychiatric conditions, including TBI sequelae. A widely used technique is transcranial direct current stimulation (tDCS), which has the potential to modify and modulate the polarity of the neuron’s membrane current. tDCS has been tested in some diseases including TBI. Its principle is based on the application of a low intensity electrical current that flows from the anode to the cathode to modify the resting potential of the membrane and modulate the level of activity of spontaneous excitatory neurons through two electrodes positioned on the individual’s scalp (Fregni and Pascual-Leone, 2007).

Research has demonstrated positive results from the application of tDCS after stroke, such as improved motor and cognitive function, when tDCS was combined with other therapies. Other studies have mainly explored the use of non-invasive brain stimulation on psychiatric disorders and in healthy individuals (for performance evaluation). However, previous studies have failed to report conclusive evidence to support or refute the use of tDCS after TBI (Hong-Yu et al., 2023; González-Rodriguez et al., 2022). Factors related to biological systems and individual variability are the main reasons underlying some of these inconsistencies. Studies showing positive results from tDCS after TBI were carried out on individuals in the chronic phase of trauma (Yan et al., 2020), revealing a lack of studies examining tDCS in the acute phase of TBI, which would be clinically relevant as studies suggest that early interventions are optimal for optimal recovery. According to Zaninotto et al. (2019) combining tDCS with cognitive and/or physical training can increase long-term potentiation (LTP). In view of the above, there is a need to carry out more studies in the acute or subacute phase of TBI since, to date, there is no publication of studies in these initial phases using non-invasive brain stimulation (tDCS).

Therefore, the objective of this study is to bring clinical case reports of patients with severe traumatic brain injury (TBI) who underwent tDCS in the sub-acute phase. The investigation sought to understand the therapeutic effects of tDCS on the functional and cognitive recovery of these patients, identify possible variations in results depending on the individual characteristics of the patients and the stimulation protocol used, and evaluate the safety and tolerability of the method. Furthermore, the study aims to contribute to the development of evidence-based clinical guidelines for the application of tDCS in patients with TBI.

## Case description

This is a prospective, single-group forecasting study with a pre-test, post-test and follow-up, which follows the CONSORT (Consolidated Reporting Standards) extension for planned pilot testing and forecasting.

The sample consisted of 5 men with a mean age of 35.2 ± 18.75, admitted to the State Hospital for Urgency and Emergency, located in the city of Vitória, Espirito Santo, Brazil.

Patients underwent screening, which included anamnesis and assessment of inclusion and exclusion criteria.

Participants were included if they: had severe TBI, had a Glasgow Coma Scale (GCS) score ≤ 8 on admission or at some point during hospital stay, were in the subacute phase of the trauma (between 2 and 16 weeks), were between 15 and 80 years old.

They were excluded if they had: craniectomy, cranioplasty, previous neurological diseases, epidural hematoma, epilepsy and pain or bedsores that prevented the application of tDCS or that did not voluntarily accept to participate in the research.

## Treatment

**Figure 1:**
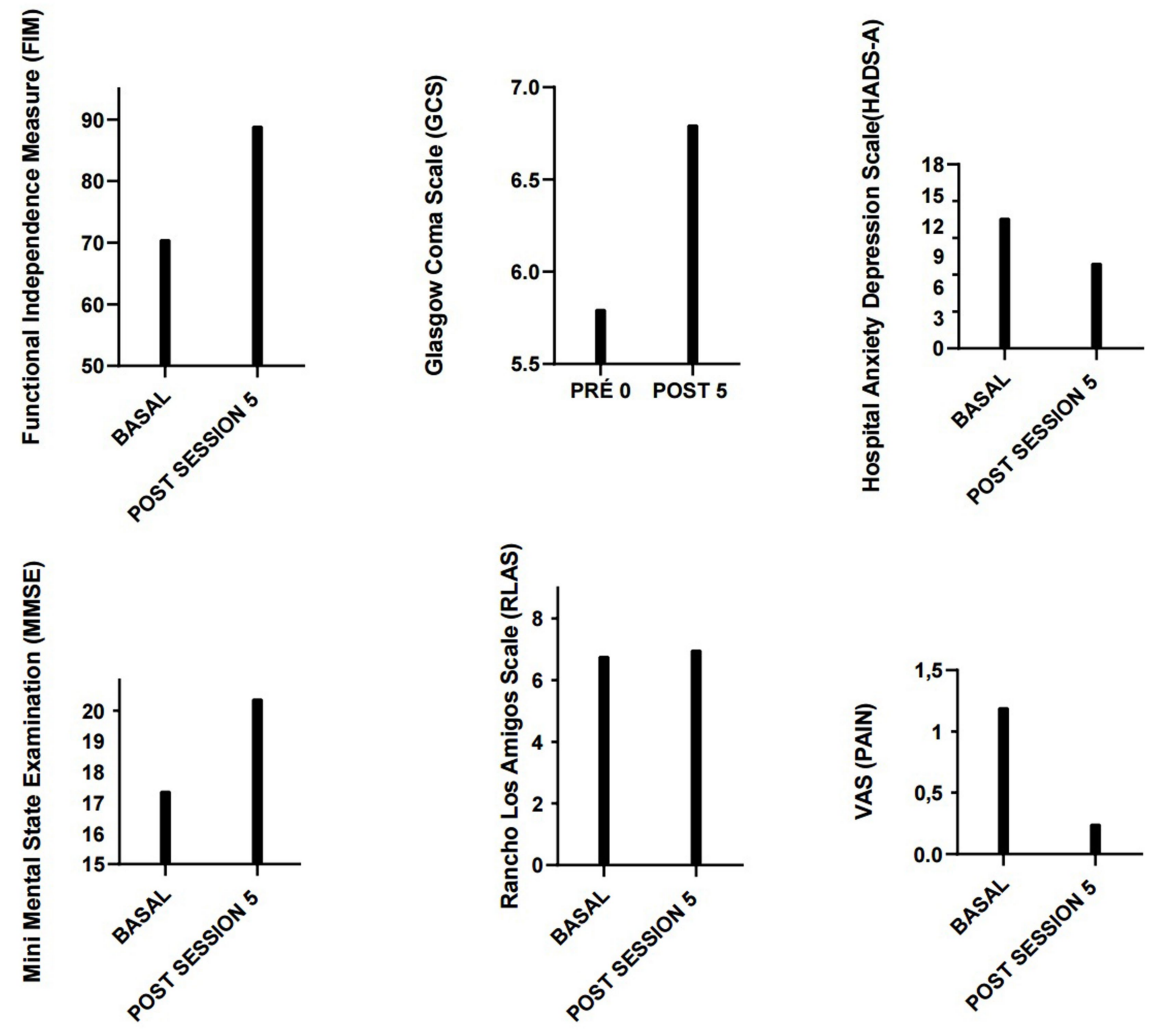
Results.

Participants received 5 tDCS sessions every day. The results were measured at the beginning and end of the 5 sessions. The application of tDCS was with an intensity of 2 mA. The Stimulator (DC Plus stimulator, Neuroconn, Ilmenau, Germany) provided direct current through a pair of electrodes. Surface (electrode size 35 cm2), spongy, soaked in 0.9% saline solution. The active electrode (anode) was applied to the region of the left dorsolateral prefrontal cortex (CPFDLE - F3) based on the study of Kolskar (Kolskår et al., 2021) according to the international system 10–20 and the cathode was positioned over the contralateral supraorbital area (Figure 1).

Clinical outcomes were measured using cognitive assessment, the Mini Mental State Examination (MMSE), mental health and depression, the Hospital Anxiety and Depression Scale (HADS-A), MMSE scores can be influenced by factors such as educational level, age, language, and ethnicity (Crum et al., 1993; Scazufca et al., 2009). Furthermore, the ability of the MMSE to identify patients with mild cognitive impairment has been considered limited (Pendlebury et al., 2010). Based on this, we used another cognitive test, the Hospital Anxiety and Depression Scale (HADS), which is widely used in clinical research. In addition, it is important to include assessments of patients’ mental state regarding symptoms of anxiety and depression, which can provide a more comprehensive view of their clinical condition.

In addition, we assessed pain, using the visual analogue scale (VAS-PAIN), Functional Independence Measure (FIM). In addition to the study results, two scales, Rancho Los Amigos Scale (RLAS) and Glasgow Outcome Scale-Extended (GOS-E), were applied to classify the patient’s condition.

The study obtained ethical approval from the Research Ethics Committee (CAAE32061920.6.0000.5060) of the Federal University of Espírito Santo, Vitória, Brazil. All study procedures remain in accordance with the Declaration of Helsinki. Informed consent was obtained.

## Results

Due to the nature of an estimation study, no formal sample size calculation was performed. And, it did not have the statistical power to detect clinically significant differences in the progress assessed. For characteristics of participants and findings results, descriptive statistics were presented as mean ± Standard Deviation (SD).

Patients were assessed using scales before and after 5 days of tDCS intervention. The average evaluation results are presented below. Data reveals significant mean improvements across all scales assessed following tDCS intervention:

Mini Mental State Examination (MMSE): The average score increased from 17.2 to 20.4, indicating an improvement in cognitive function.

Hospital Anxiety and Depression Scale - Anxiety Subscale (HADS-A): The average score decreased from 12.8 to 8.4, suggesting a reduction in anxiety levels.

Visual Analogue Scale (VAS): The average score decreased from 1.2 to 0.25, indicating a reduction in pain perception.

Functional Independence Measure (FIM): The average score increased from 70.6 to 89, reflecting an improvement in patients’ functional independence.

Rancho Los Amigos Scale (RLAS): The average score increased slightly from 6.8 to 7, suggesting a small improvement in cognitive and behavioral responsiveness.

Glasgow Outcome Scale - Extended (GOS-E): The average score increased from 5.8 to 6.8, indicating an improvement in the patients’ overall outcome.

## Discussion

In a sample of five patients, we tested the effect of tDCS in patients with sub-acute TBI. To our knowledge, no prior studies have examined effects of tDCS on sub-acute patients with TBI.

The results observed after the tDCS intervention, such as the increase in the MMSE score, which suggests an improvement in the patients’ cognitive abilities, were previously described by Wang et al. (2022). The substantial improvement in the evaluated areas, as observed in this study, corroborates the previously reported findings, reinforcing the effectiveness of the intervention.

The reduction in HADS-A and VAS scores indicates a significant decrease in anxiety levels and pain perception, respectively, suggesting an improvement in overall quality of life. As observed in this study, these findings are consistent with previous research, further reinforcing the effectiveness of the intervention (Starkstein and Hayhow, 2019).

The notable improvement in the FIM score points to an increase in patients’ functional independence, which is crucial for rehabilitation (Dong et al., 2021). Although improvements in the RLAS and GOS-E scales are less pronounced, they still indicate a positive trend in patient recovery.

Direct comparison between studies is complicated due to differences in protocols, regarding both electrode montage and location (both in terms of the cathode and anode), stimulation frequency, number of sessions, and current amperage. There might be greater treatment benefit with higher number of sessions and increased stimulation intensity (Charvet et al., 2018).

Some preliminary studies have shown beneficial effects of tDCS on cognitive function in healthy individuals as well as in stroke patients and these studies demonstrate good tolerability and safety in patients (Kazuta et al., 2017). Moreover, tDCS treatment response may interact with individual characteristics such as time since stroke onset, lesion location, or lesion size.

## Conclusion

This study investigated the effects of transcranial direct current stimulation (tDCS) in patients with subacute traumatic brain injury (TBI), exploring this approach in patients with this specific condition. The results found suggest that tDCS may have a positive impact on the recovery of these patients, evidenced by the improvement in all assessed scores.

Although there is little evidence on the effects of tDCS in acute stroke patients, the results of this study suggest that tDCS may be a promising intervention, with the potential to benefit the recovery of patients with subacute TBI. However, it is essential to consider individual patient characteristics, such as time since injury onset, location and size of the lesion, when planning tDCS treatments.

Future studies with larger sample sizes and long-term follow-ups are needed to confirm these results and better understand the neurophysiological mechanisms underlying the observed improvements.

The positive results observed may guide healthcare professionals to adopt similar transcranial direct current stimulation (tDCS) protocols in their clinical practices. This can standardize treatments and improve outcomes for TBI patients. Furthermore, this research provides a basis for future studies that could explore different parameters of tDCS, such as duration and intensity of stimulation, as well as its combination with other therapeutic interventions.

### Strength and limitation

Our case report highlights several strengths, including be first study to explore tDCS in the subacute phase of TBI, offering potential for early intervention, use of multiple outcome measures (cognitive, functional, psychological). Have clinical relevance conducted in a real-world hospital setting, making the results applicable to clinical practice. However, it does have some limitations, such short intervention duration, only five tDCS sessions, which may not capture long-term effects, lack of statistical power to detect significant differences, requiring further research to confirm findings.

## Data Availability

The datasets presented in this article are not readily available because requests to access the datasets should be directed to fernandozanela@hotmail.com.
